# Resistin: A Potential Indicator of Aortic Stiffness in Non-Dialysis Chronic Kidney Disease Patients

**DOI:** 10.3390/medicina59091652

**Published:** 2023-09-13

**Authors:** Chiu-Huang Kuo, Min-Shuo Chen, Chih-Hsien Wang, Yu-Hsien Lai, Yu-Li Lin, Bang-Gee Hsu

**Affiliations:** 1 Division of Nephrology, Hualien Tzu Chi Hospital, Buddhist Tzu Chi Medical Foundation, Hualien 97004, Taiwan; hermit.kuo@gmail.com (C.-H.K.); nomo8931126@gmail.com (Y.-L.L.); 2 School of Post-Baccalaureate Chinese Medicine, Tzu Chi University, Hualien 97004, Taiwan; 3 School of Medicine, Tzu Chi University, Hualien 97004, Taiwan

**Keywords:** aortic stiffness, carotid–femoral pulse wave velocity, resistin, non-dialysis-dependent chronic kidney disease

## Abstract

*Background and Objectives*: In the progression and development of atherosclerosis, resistin plays a significant role. Chronic kidney disease (CKD), frequently associated with atherosclerosis, exhibits a marked increase in morbidity and mortality rates. This study set out to explore the association between aortic stiffness and serum levels of resistin in non-dialysis-dependent CKD patients ranging from stages 3 to 5. *Materials and Methods*: We collected fasting blood samples from 240 CKD patients across stages 3 to 5. The concentration of resistin in serum was determined using a commercially available enzyme immunoassay kit. Those patients who exhibited a carotid–femoral pulse wave velocity (cfPWV) greater than 10 m/s were identified as the aortic stiffness group. *Results*: Out of the 240 CKD patients, 88 (36.7%) were classified within the aortic stiffness group. This group demonstrated higher incidences of diabetes, advanced age, increased body weight, body mass index, body fat mass, systolic and diastolic blood pressure, fasting glucose, and serum resistin levels. Multivariate logistic regression analysis highlighted resistin, diabetes, and body weight as independent predictors of aortic stiffness. Additionally, body fat mass, logarithmically transformed cfPWV (log-cfPWV) values and log-triglyceride levels were independent predictors of log-resistin levels by multivariate stepwise linear regression analysis. *Conclusions*: In CKD patients from stages 3 to 5, a positive correlation exists between elevated serum resistin levels and cfPWV values, identifying resistin as a potential predictor of aortic stiffness.

## 1. Introduction

Arterial stiffening yields several hemodynamic implications, including an expansion of pulse pressure, a decrease in shear stress rate, and an enhanced transmission of pulsatile flow into the microcirculation [1]. Ample studies have established carotid-femoral pulse wave velocity (cfPWV) as a robust tool for measuring aortic stiffness, with strong predictive power for cardiovascular (CV) morbidity and mortality [2,3,4]. Notably, patients with chronic kidney disease (CKD) have been observed to exhibit a higher incidence of CV mortality compared to the development of end-stage renal disease (ESRD) [5]. Atherosclerosis, a leading cause of CV-related mortality in CKD, is frequently accompanied by an increase in aortic stiffness [6]. Consequently, the imperative nature of predicting CV risk in CKD patients has gained significant recognition. The value of cfPWV measurements extends beyond traditional and renal CV risk factors, offering additional prognostic insight for CKD patients [7].

Resistin, initially associated with obesity and insulin resistance in rodent models, has been identified as a macrophage-derived secretory product in humans and is implicated in both inflammation and insulin resistance [8]. Prior research has shown resistin to be a harbinger of atherosclerosis, seemingly implicated in its pathogenesis via vascular endothelial dysfunction, vascular smooth muscle cell (VSMC) proliferation, and foam cell transformation [9]. Our preceding research has further indicated that serum resistin levels could serve as a potential biomarker for aortic stiffness in patients with coronary artery disease (CAD) [10]. Importantly, resistin levels have recently been shown also to be elevated in CKD patients and could be associated with their higher risk of cardiovascular events [11].

Considering that aortic stiffness is a well-established predictor of CV mortality and that resistin is associated with both atherosclerosis and CKD has led us to hypothesize that resistin might play a crucial role in predicting aortic stiffness in CKD patients. Therefore, in this study, we specifically examined the correlation between serum resistin levels and aortic stiffness in patients with non-dialysis-dependent CKD.

## 2. Materials and Methods

### 2.1. Enrollees

In a study conducted from January to December 2016, subjects were recruited from the renal outpatient department of a medical center in Hualien, Taiwan. The research conducted herein has been reviewed and granted ethical approval by the Research Ethics Committee, Hualien Tzu Chi Hospital, adhering to the ethical standards set forth for research involving human subjects. All participants, who were over the age of 18, provided written informed consent. Patients involved in the study underwent multidisciplinary CKD care, which included avoidance of nephrotoxins and dietary restrictions on salt and protein. Diagnosis of CKD was based on two estimated glomerular filtration rate (eGFR) readings, computed using the CKD-EPI (Chronic Kidney Disease Epidemiology Collaboration) equation, taken three months apart [12]. eGFR values below 60 mL/min per 1.73 m^2^ were classified as CKD. The subjects were then categorized into different CKD stages per the Kidney Disease Outcomes Quality Initiative criteria [13]. For inclusion in the study, participants were required to meet specific criteria. These included being aged over 18 years, providing written informed consent, having a diagnosis of CKD based on eGFR values below 60 mL/min per 1.73 m^2^, and undergoing multidisciplinary CKD care. On the other hand, participants were excluded from the study if they had existing malignancies, chronic inflammatory diseases, heart failure, or chronic obstructive pulmonary disease at the time of blood collection, or if they were scheduled to commence renal replacement therapy within the upcoming six months.

### 2.2. Physical Measurements

With body weight and height, the body mass index (BMI) was derived using Quetelet’s formula, dividing weight in kilograms by the square of height in meters. Body fat mass was gauged using a standard tetrapolar bioimpedance technique for the whole body (hand–foot), facilitated by a single-frequency (50 kHz) analyzer (Biodynamic-450, Biodynamics Corporation, Seattle, WA, USA) [10,14,15].

### 2.3. Biochemical Investigations

After overnight fasting, blood samples were collected for routine chemistry measurements, and a commercially available enzyme immunoassay (EIA) kit was used to measure human serum resistin concentrations. (SPI-BIO, Montigny le Bretonneux, France) [10,14,15].

### 2.4. Carotid–Femoral Pulse Wave Velocity Measurements

To assess aortic stiffness, cfPWV values were determined using pressure applanation tonometry (SphygmoCor system, AtCor Medical, Sydney, NSW, Australia) [10]. Participants did not halt their regular medication during the assessment of cfPWV values. These measurements were conducted in a temperature-controlled, quiet room, with the subject in a supine position following at least 10 min of rest. An R-timing interval was provided by a simultaneous ECG monitor. After 10 min of rest, pulse wave recordings were performed on the carotid and femoral artery region. Records were taken concurrently with ECG signals for R–R timing interval. The distance between the carotid and femoral locations was determined by subtracting the distance from the carotid location to the suprasternal notch from the distance between the suprasternal and femoral sites. Integral software calculated the mean time difference between the R-wave and pulse wave on a beat-to-beat basis for each pulse wave and ECG data set, over ten consecutive cardiac cycles. Following the standards of the European Society of Hypertension (ESH) and the European Society of Cardiology (ESC), subjects exhibiting a cfPWV exceeding 10 m/s were classified as part of the aortic stiffness group [16].

### 2.5. Statistical Analysis

The Kolmogorov–Smirnov test was applied to assess the normality of the continuous variables. Depending on the Gaussian distribution, data were either presented as mean ± standard deviation or as a median accompanied by the interquartile range (IQR). The Student’s independent *t*-test or the two-tailed Mann–Whitney U test was employed to draw comparisons between the aortic stiffness and non-aortic stiffness groups, as deemed fit. Categorical data, represented as counts and percentages, were evaluated using the χ^2^ test. Multivariable logistic regression analysis was used to determine the independence of variables significantly associated with aortic stiffness in patients with CKD. Nonnormally distributed continuous variables were converted to the natural logarithm (log) scale to facilitate linear regression analysis. Through the use of simple and multivariate linear regression analysis, the relationships between log-resistin levels and other variables were evaluated. The threshold for statistical significance was set at a *p*-value less than 0.05. All statistical procedures were conducted utilizing the SPSS software (version 24).

## 3. Results

### 3.1. Baseline Characteristics of This Study

We initially enrolled 295 patients from the clinic, out of which 55 patients were excluded. Of these exclusions, 20 had heart failure, 10 had a history of malignancy, 12 had COPD, 5 had acute infections, and 8 failed to complete the examination. This left us with a cohort of 240 eligible patients for analysis. The clinical attributes of the 240 patients with CKD under study are presented in Table 1, which reveals comorbidities, including diabetes mellitus (DM; *n* = 107; 44.6%), hypertension (*n* = 198; 82.5%), and chronic glomerulonephritis (*n* = 53; 22.1%). Within this cohort, aortic stiffness was detected in 36.7% (*n* = 88) of patients. The prevalence of aortic stiffness was markedly higher among CKD patients with DM as opposed to those without DM (*p* = 0.018). Gender, comorbidity with hypertension, or chronic glomerulonephritis did not exhibit a statistically significant difference between the two groups. Notably, the group exhibiting aortic stiffness was older (*p* < 0.001) and displayed elevated body weight (*p* = 0.001), BMI (*p* = 0.002), body fat mass (*p* = 0.002), systolic blood pressure (SBP; *p* < 0.001), diastolic blood pressure (DBP; *p* = 0.004), fasting glucose (*p* = 0.009), and serum resistin level (*p* < 0.001) when contrasted with the non-aortic stiffness group.

### 3.2. Resistin Level Is Associated with Aortic Stiffness in Patients with CKD

From these results, serum resistin levels (OR: 1.090, 95% CI: 1.032–1.152, *p* = 0.002), DM (OR: 2.373, 95% CI: 1.161–4.852, *p* = 0.018), age (OR: 1.063, 95% CI: 1.031–1.095, *p* < 0.001), and body weight (OR: 1.057, 95% CI: 1.009–1.007, *p* = 0.019) surfaced as independent predictors of aortic stiffness in non-dialysis-dependent CKD patients, revealing the intricacies of the aortic stiffness-related factors as per our multivariable logistic regression analysis (Table 2).

### 3.3. Correlation between Resistin Levels and Clinical and Biochemical Parameters in CKD Patients

Finally, as we examined the correlation matrix between serum log-resistin levels and clinical variables in CKD patients, as illustrated in Table 3; we noticed that body weight (r = 0.244, *p* < 0.001), BMI (r = 0.305, *p* < 0.001), body fat mass (r = 0.422, *p* < 0.001), and log-cfPWV values (r = 0.249, *p* < 0.001) were positively correlated with resistin levels. Meanwhile, log-triglycerides exhibited a negative correlation (r = −0.134; *p* = 0.037). The final revelation of this study came from the multivariate stepwise linear regression analysis, which unveiled that body fat mass (β = 0.401; adjusted R^2^ change = 0.192, *p* < 0.001), log-cfPWV values (β = 0.204; adjusted R^2^ change = 0.031, *p* < 0.001), and log-triglyceride level (β = −0.130; adjusted R^2^ change = 0.013, *p* = 0.024) were independent predictors of log-resistin levels in subjects with CKD before dialysis.

## 4. Discussion

The principal finding of our study is a high prevalence of DM, advanced age, elevated body weight, BMI, body fat mass, SBP, DBP, fasting glucose, and serum resistin levels among non-dialysis-dependent CKD patients with aortic stiffness. We determined a positive correlation between serum log-resistin levels and log-cfPWV values, positioning resistin as an independent predictor of aortic stiffness in this patient cohort. Additionally, body fat mass and serum log-triglyceride levels emerged as independent predictors of log-resistin values in these patients.

Resistin is significantly expressed in human monocytes and macrophages and has been associated with obesity, insulin resistance, and diabetes, as well as contributing to cardiovascular disease development. It has been found that resistin levels show a positive correlation with fat mass, insulin resistance, and interleukin 6 (IL-6). Resistin is induced by genetic factors and several stimuli, leading to the inhibition of insulin receptor signaling, and subsequently causing insulin resistance [17]. In a study by López et al., it was observed that overweight and obese individuals exhibited higher plasma resistin concentrations compared to those in a lower BMI range (18.5 kg/m^2^ to 24.99 kg/m^2^) [18]. Moreover, resistin showed a positive correlation with body fat percentage. The study also proposed that resistin might drive the accumulation of cholesterol and triglycerides in macrophages through glucose-dependent mechanisms [19]. Expanding on this, serum resistin has been observed to stimulate the proliferation of human aortic smooth muscle cells and promote VSMC migration [8]. In patients with coronary artery disease, serum resistin has emerged as an independent marker of aortic stiffness [10]. These findings suggest that resistin could play a crucial role in predicting CV risks.

Turning to our own study involving non-dialysis-dependent CKD patients in stages 3 to 5, we found that serum resistin levels correlated positively with body fat mass, body weight, and BMI, and inversely with log-triglyceride levels. Furthermore, body fat mass and log-triglyceride levels emerged as independent predictors of log-resistin values in a multivariable stepwise linear regression analysis, providing novel insights in this disease context. The crucial inference from our study is that serum resistin levels hold significant promise in predicting the development of aortic stiffness in CKD stage 3 to 5 patients. This finding not only enhances our comprehension of CKD pathophysiology, but also suggests the possibility of early intervention strategies for preventing cardiovascular complications in CKD patients, further solidifying the critical role resistin could play in clinical prognostics.

Pivoting to the mechanism of aortic stiffness, this condition stems from a complex interplay involving vascular smooth muscle cells and the extracellular matrix, comprised of elastin, collagen, and fibrillin fibers. The forward propulsion of blood in the aorta is facilitated by elastic recoil. However, mechanical stress, such as that resulting from aging, can induce aortic stiffness [20]. As the arterial system ages, degradation of elastic fibers and endothelial dysfunction ensue, giving rise to arterial stiffness [1,2]. This stiffness in turn elevates SBP, reduces coronary perfusion, and intensifies pulse load on the microcirculation [21]. Building on these understandings, our study identifies an association between aortic stiffness and increased age, SBP, and DBP among CKD patients.

In the context of diabetes, hyperglycemia catalyzes the production of advanced glycation end products (AGEs) through a series of reactions between reducing sugars and proteins [22,23]. AGEs not only contribute to diabetes complications, such as atherosclerosis and small vessel disease, but also accelerate smooth muscle cell calcification by promoting osteoblast-like differentiation [24]. Diabetes could exacerbate aortic stiffness by damaging and depleting elastic fibers, in addition to promoting the proliferation of collagen fibers in the aortic media [25]. Wohlfahrt et al. observed that patients with a normal BMI but increased body fat were more prone to metabolic disturbances and exhibited higher cardiovascular mortality [26]. They posited that increased aortic stiffness intensifies cardiac workload and pressure and leads to unstable flow in the cerebral and renal microcirculation, thereby causing microvascular injury [27]. In line with these findings, our study provides compelling evidence that CKD stage 3 to 5 patients in the aortic stiffness group have higher prevalence of diabetes, body fat mass, and BMI. These findings underscore the role of aortic stiffness as an important clinical parameter in CKD patients.

On the progression of CKD, it is associated with significantly heightened CV mortality [28,29]. A decrease in the estimated glomerular filtration rate (eGFR) and increased albuminuria have been independently linked to the onset of myocardial infarction, stroke, CV disease mortality, and all-cause mortality [28]. A decline in renal function has been shown to be linked with aortic stiffness and elevated cfPWV values [30]. Huang et al. substantiated these findings by demonstrating a correlation between higher cfPWV values and declining eGFR [31]. It is hypothesized that the decrease in eGFR accompanying aortic stiffness results from excessive fluctuating flow, which may reduce cortical artery volume, augment renal vascular resistance, and decrease function by impairing muscle tone or remodeling. This series of events could precipitate renal microcirculatory injury and loss of glomeruli and vessels [32]. Additional potential mechanisms connecting aortic stiffness with renal dysfunction may include chronic inflammation, oxidative stress, and activation of the renin–angiotensin system [30]. In patients with CAD, hypertension, and type 2 DM, resistin is negatively associated with eGFR [10,15,33]. Our study did not find that renal function or CKD stage exhibit a statistically significant difference between the aortic stiffness and non-aortic stiffness groups. Moreover, there were no statistically significant differences in serum log-resistin level and renal function in CKD stage 3 to 5 patients. Further study in larger CKD cohorts is required to confirm the relationship between renal function and aortic stiffness or resistin level in CKD patients.

This study does have several limitations that warrant consideration. As a cross-sectional study conducted in a single center, the results might not be generalizable to other populations. Additionally, it is well-acknowledged that pharmacological interventions can influence human cfPWV or resistin levels. For instance, previous studies have documented a greater reduction in cfPWV values following treatment with a combination of olmesartan and azelnidipine compared to a regimen combining olmesartan and hydrochlorothiazide [34]. Furthermore, a significant decrease in daily mean plasma resistin concentrations was observed after a 6-week course of treatment with amlodipine, bisoprolol, or indapamide [35]. The CKD subjects in our study widely used these anti-hypertensive agents, which could have affected serum resistin concentrations. However, we did not document the specific anti-hypertensive medications utilized, nor did we explore the potential association between these drugs and cfPWV values. Further investigation is required to establish a definitive link between serum resistin levels and aortic stiffness in patients with CKD.

## 5. Conclusions

In conclusion, this study discovered an association between elevated resistin levels and aortic stiffness in patients with non-dialysis-dependent CKD stage 3 to 5. Our results also revealed a positive relationship between serum log-resistin levels and log-cfPWV values within the same patient population. These findings suggest that serum resistin levels may be a significant predictor of aortic stiffness in non-dialysis-dependent CKD patients. To fully comprehend the implications of these results, additional research is necessary to elucidate the underlying mechanisms linking serum resistin levels and aortic stiffness.

## Figures and Tables

**Table 1:** Summary of Participant Characteristics in Aortic Stiffness and Non-Aortic Stiffness Groups.

Clinical Attributes	All Patients (*n* = 240)	Non-Aortic Stiffness Group (*n* = 152)	Aortic Stiffness Group (*n* = 88)	*p*-Value
Age (years)	68.85 ± 13.66	66.18 ± 14.10	73.47 ± 11.55	<0.001 *
Height (cm)	158.63 ± 8.56	158.11 ± 8.30	159.54 ± 8.97	0.211
Body weight (kg)	65.74 ± 14.26	63.45 ± 13.31	69.71 ± 15.05	0.001 *
BMI (kg/m^2^)	26.02 ± 4.65	25.31 ± 4.55	27.24 ± 4.57	0.002 *
Body fat mass (%)	28.90 ± 8.86	27.65 ± 9.07	31.22 ± 8.06	0.002 *
cfPWV (m/s)	9.05 (7.20–11.28)	7.70 (6.53–8.90)	12.45 (10.83–14.45)	<0.001 *
SBP (mmHg)	149.98 ± 24.90	145.24 ± 24.18	158.17 ± 24.11	<0.001 *
DBP (mmHg)	84.35 ± 13.37	83.01 ± 12.74	86.68 ± 14.178	0.004 *
TCH (mg/dL)	159.91 ± 41.63	160.08 ± 44.59	159.63 ± 36.21	0.935
Triglyceride (mg/dL)	122.0 (91.00–170.00)	118.50 (87.25–164.00)	135.50 (95.75–188.00)	0.063
Fasting glucose (mg/dL)	99.00 (90.00–126.75)	97.00 (89.00–124.50)	107.00 (94.25–134.50)	0.009 *
BUN (mg/dL)	33.50 (24.25–50.00)	33.00 (23.25–51.75)	35.00 (26.25–47.00)	0.410
Creatinine (mg/dL)	1.95 (1.40–2.90)	1.90 (1.40–2.88)	2.05 (1.50–3.05)	0.346
eGFR (mL/min)	30.80 ± 15.56	31.90 ± 16.26	28.92 ± 14.15	0.154
Total calcium (mg/dL)	8.88 (8.60–9.24)	8.86 (8.56–9.28)	8.96 (8.68–9.20)	0.594
Phosphorus (mg/dL)	3.70 (3.20–4.28)	3.70 (3.23–4.30)	3.70 (3.10–4.20)	0.433
Resistin (ng/mL)	5.17 (3.31–8.21)	7.39 (5.89–11.73)	10.70 (7.14–18.85)	<0.001 *
Female, *n* (%)	110 (45.8)	76 (50.0)	34 (38.6)	0.089
Diabetes mellitus, *n* (%)	107 (44.6)	59 (38.8)	48 (54.5)	0.018 *
Hypertension, *n* (%)	198 (82.5)	127 (83.6)	71 (80.7)	0.573
Glomerulonephritis, *n* (%)	53 (22.1)	39 (25.7)	14 (15.9)	0.079
CKD stage 3, *n* (%)	116 (48.3)	77 (50.7)	39 (44.3)	0.538
CKD stage 4, *n* (%)	72 (30.0)	42 (27.6)	30 (34.1)	
CKD stage 5, *n* (%)	52 (21.7)	33 (21.7)	19 (21.9)	

Values for continuous variables are given as mean ± standard deviation and tested by Student’s *t*-test; variables not normally distributed are given as median and interquartile range and tested by Mann–Whitney U test; values are presented as number (%) and analysis was done using the chi-square test. BMI, body mass index; cfPWV, carotid–femoral pulse wave velocity; SBP, systolic blood pressure; DBP, diastolic blood pressure; TCH, total cholesterol; BUN, blood urea nitrogen; eGFR, estimated glomerular filtration rate. * *p* < 0.05 was considered statistically significant.

**Table 2:** Multivariate logistic regression analysis of the factors correlated to aortic stiffness.

Variables	Odds Ratio	95% C.I.	*p*-Value
Resistin, 1 ng/mL	1.090	1.032–1.152	0.002 *
Age, 1 year	1.063	1.031–1.095	<0.001 *
Diabetes mellitus, present	2.373	1.161–4.852	0.018 *
Body weight, 1 kg	1.057	1.009–1.107	0.019 *
Systolic blood pressure, 1 mmHg	1.015	0.995–1.036	0.140
Diastolic blood pressure, 1 mmHg	1.015	0.977–1.054	0.448
Body fat mass, 1%	1.032	0.987–1.079	0.170
Body mass index, 1 kg/m^2^	0.884	0.759–1.029	0.112
Fasting glucose, 1 mg/dL	1.001	0.993–1.008	0.856

Multivariate logistic regression analysis was employed for data analysis, incorporating factors such as diabetes mellitus, age, body weight, body fat mass, body mass index, systolic blood pressure, diastolic blood pressure, fasting glucose, and resistin. The statistical significance was determined at * *p* < 0.05, and confidence intervals (CI) were calculated.

**Table 3:** Correlation between serum logarithmically transformed resistin levels and clinical variables.

Variables	Log-Resistin (ng/mL)				
	Simple Regression		Multivariable Regression		
	r	*p*-Value	Beta	Adjusted R^2^ Change	*p*-Value
Female	0.009	0.885	—	—	—
Diabetes mellitus	−0.019	0.771	—	—	—
Hypertension	−0.075	0.245	—	—	—
Glomerulonephritis	0.032	0.619	—	—	—
Age (years)	0.120	0.064	—	—	—
Height (cm)	−0.026	0.686	—	—	—
Body weight (kg)	0.244	<0.001 *	—	—	—
BMI (kg/m^2^)	0.305	<0.001 *	—	—	—
Body fat mass (%)	0.442	<0.001 *	0.401	0.192	<0.001 *
Log-cfPWV (m/s)	0.249	<0.001 *	0.204	0.031	<0.001 *
SBP (mmHg)	0.124	0.054	—	—	—
DBP (mmHg)	0.102	0.115	—	—	—
TCH (mg/dL)	−0.023	0.719	—	—	—
Log-Triglyceride (mg/dL)	−0.134	0.037 *	−0.130	0.013	0.024 *
Log-Glucose (mg/dL)	0.058	0.367	—	—	—
Log-BUN (mg/dL)	0.027	0.679	—	—	—
Log-Creatinine (mg/dL)	0.024	0.710	—	—	—
eGFR (mL/min)	−0.051	0.431	—	—	—
Log-Calcium (mg/dL)	0.012	0.852	—	—	—
Log-Phosphorus (mg/dL)	−0.053	0.410	—	—	—

Data on carotid–femoral pulse wave velocity, triglyceride, glucose, BUN, creatinine, calcium, phosphorus, and resistin levels showed skewed distribution and, therefore, were log-transformed before analysis. Data analysis was performed using the simple linear regression or multivariate stepwise linear regression analysis (adapted factors were body weight, BMI, body fat mass, log-cfPWV, and log-triglyceride). BMI, body mass index; cfPWV, carotid–femoral pulse wave velocity; SBP, systolic blood pressure; DBP, diastolic blood pressure; TCH, total cholesterol; BUN, blood urea nitrogen; eGFR, estimated glomerular filtration rate. * *p* < 0.05 was considered statistically significant.

## Data Availability

Upon request, the corresponding author can provide the data utilized in this study.
